# Supplementary material for: Predicting improved protein conformations with a temporal deep recurrent neural network
Source: PLoS One. 2018 Sep 4;13(9):e0202652. doi: 10.1371/journal.pone.0202652 (PMC6122789; doi:10.1371/journal.pone.0202652)
Supplement: S1 Fig — (A) Schematic overview of the RNN with GRU cells. (B) GRU cell, (C) & (D) Visualisation of the trajectory data ν and the process of mini-batch creation and propagation to the RNN. (PDF) [file pone.0202652.s001.pdf]

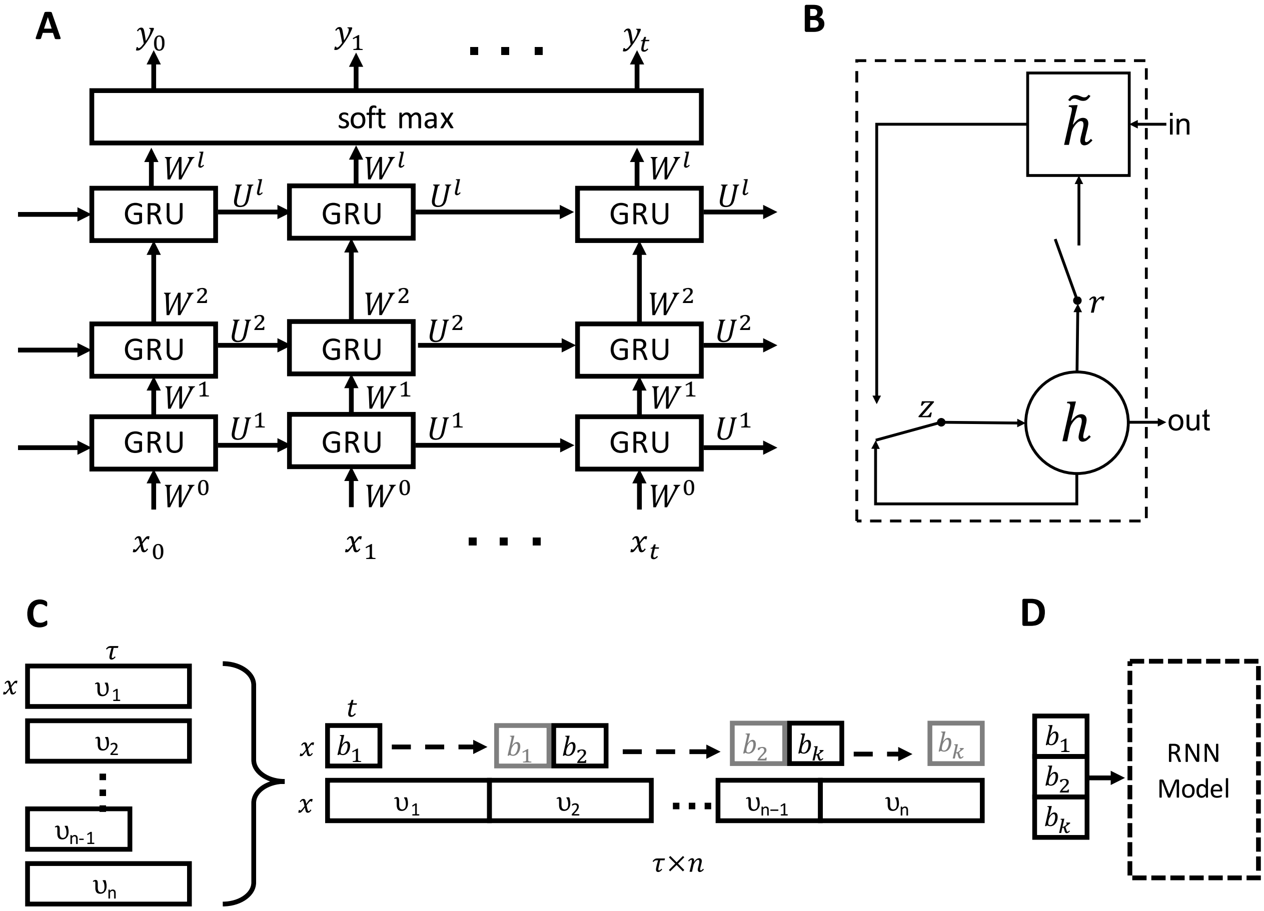

S1 Fig. : Extended DeepTrajectory method figure. (A) Schematic overview of the RNN with GRU cells. (B) GRU cell, (C) & (D) Visualisation of the trajectory data ν and the process of mini-batch creation and propagation to the RNN
