# Supplementary material for: Predicting improved protein conformations with a temporal deep recurrent neural network
Source: PLoS One. 2018 Sep 4;13(9):e0202652. doi: 10.1371/journal.pone.0202652 (PMC6122789; doi:10.1371/journal.pone.0202652)
Supplement: S2 Fig — (A) Flowchart outlining the generation of the restraints and contact maps. (B) Flowchart outlining the different MD sampling procedures (C) Example of point restraints applied to a protein. (D) Example of residue-residue distance restraints applied to a protein. (E) Definition of residue-residue contact (F) Contact map definition for CMexl and CMmin. (PDF) [file pone.0202652.s002.pdf]

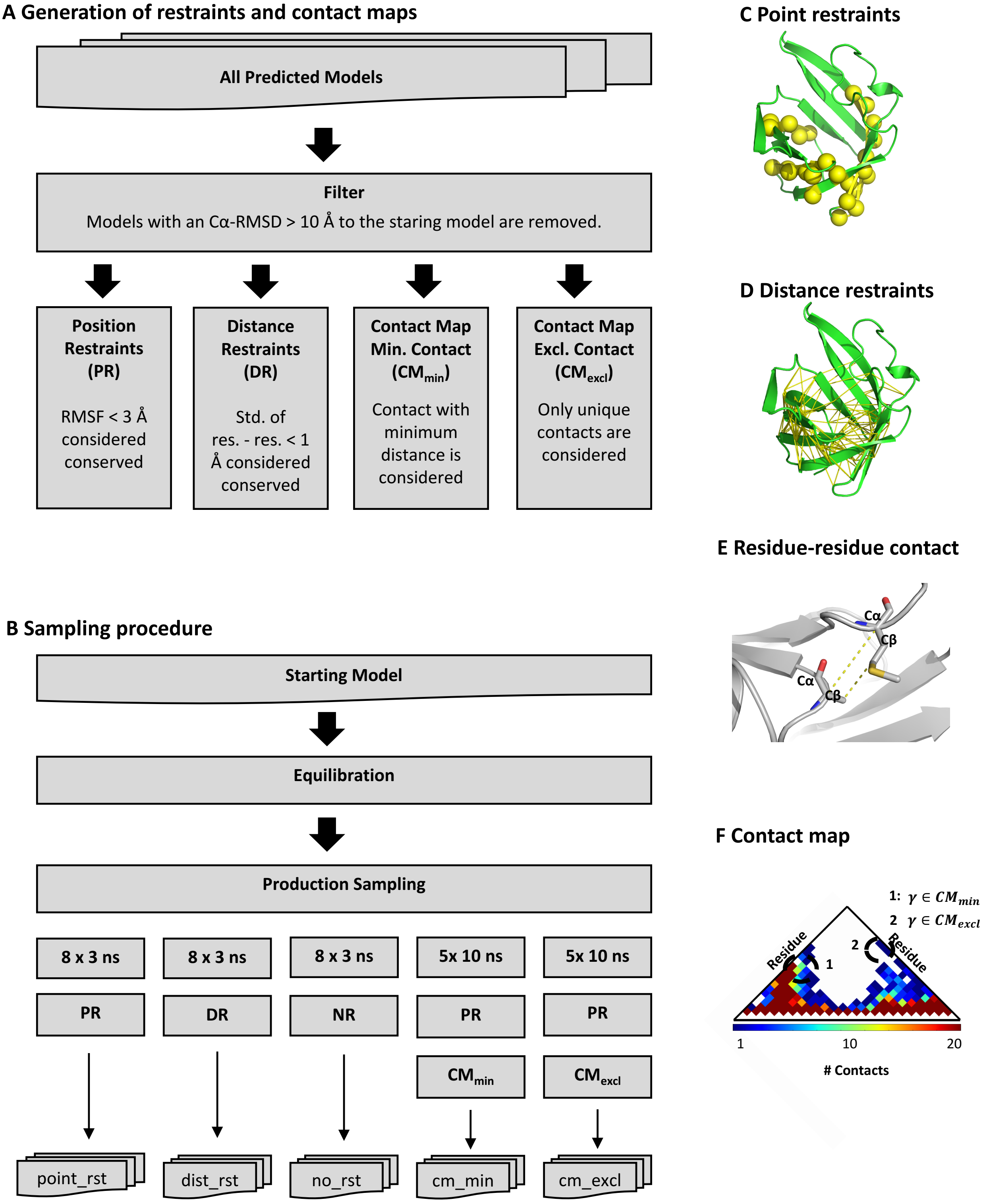

S2 Fig. : Sampling Protocol. (A) Flowchart outlining the generation of the restraints and contact maps. (B) Flowchart outlining the different MD sampling procedures (C) Example of point restraints applied to a protein. (D) Example of residue-residue distance restraints applied to a protein. (E) Definition of residue-residue contact (F) Contact map definition for CM_{exl} and CM_{min}.
