# Supplementary material for: Predicting improved protein conformations with a temporal deep recurrent neural network
Source: PLoS One. 2018 Sep 4;13(9):e0202652. doi: 10.1371/journal.pone.0202652 (PMC6122789; doi:10.1371/journal.pone.0202652)
Supplement: S3 Fig — Classification performance is shown for all three classes improved, no-change and decreased. Models with and without features RMSD_SM and GDTTS_SM are denoted as w/ dist. and w/o dist in the legend, respectively. The colored bars in purple (precision), red (recall) and green (F1) show the mean validation performance and the error bars the standard deviation of the 7-fold cross-validation. The black horizontal lines show the significance with p-value < 0.05 (*) and non-significance with p-value ≥ 0.05 (ns) between two groups as computed by the Wilcoxon rank-sum test. (PDF) [file pone.0202652.s003.pdf]

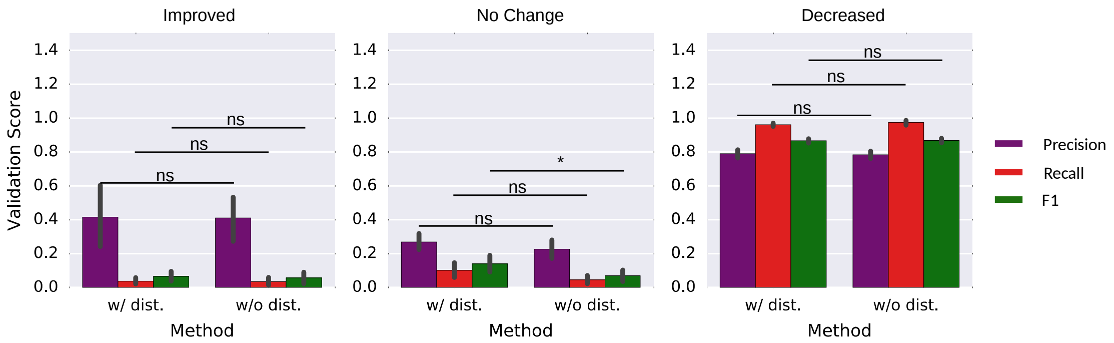

S3 Fig. : Comparison of DeepTrajectory classification performance with and without distance metric features. Classification performance is shown for all three classes improved, no-change and decreased. Models with and without features RMSD_SM and GDTTS_SM are denoted as w/ dist. and w/o dist in the legend, respectively. The colored bars in purple (precision), red (recall) and green (F1) show the mean validation performance and the error bars the standard deviation of the 7-fold cross-validation. The black horizontal lines show the significance with p-value < 0.05 (*) and non-significance with p-value ≥ 0.05 (ns) between two groups as computed by the Wilcoxon rank-sum test.
