# Supplementary material for: Predicting improved protein conformations with a temporal deep recurrent neural network
Source: PLoS One. 2018 Sep 4;13(9):e0202652. doi: 10.1371/journal.pone.0202652 (PMC6122789; doi:10.1371/journal.pone.0202652)
Supplement: S4 Fig — (A) Cumulative distribution of true positive (TP), false negative (FN) and false positive (FP) predictions for the no-change state as a function of ΔGDTTS. The background colours red, gray and green indicate the ΔGDTTS regions for the improved, no-change and decreased states, respectively. (B) Cumulative distribution of true positive (TP), false negative (FN) and false positive (FP) predictions for the no-change state as a function of GDTTS. (C) Show the distribution of assigned probabilities for no-change state predictions. (PDF) [file pone.0202652.s004.pdf]

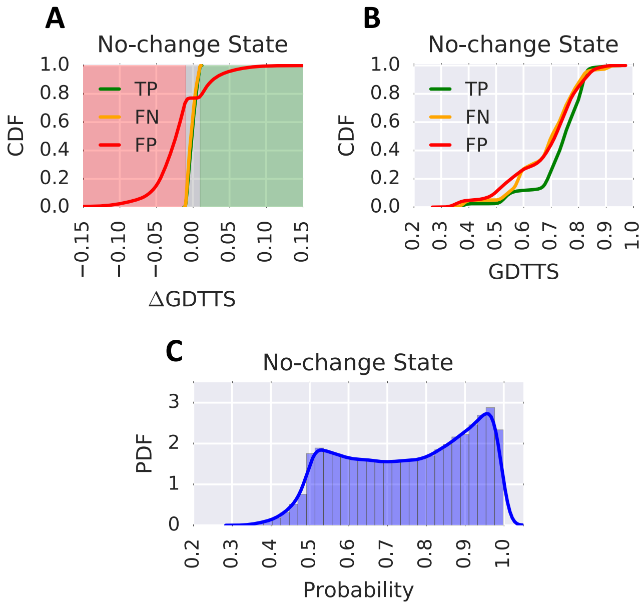

S4 Fig. : (A) Cumulative distribution of true positive (TP), false negative (FN) and false positive (FP) predictions for the no-change state as a function of ΔGDTTS. The background colours red, gray and green indicate the ΔGDTTS regions for the improved, no-change and decreased states, respectively. (B) Cumulative distribution of true positive (TP), false negative (FN) and false positive (FP) predictions for the no-change state as a function of GDTTS. (C) Show the distribution of assigned probabilities for no-change state predictions.
