# Supplementary material for: Predicting improved protein conformations with a temporal deep recurrent neural network
Source: PLoS One. 2018 Sep 4;13(9):e0202652. doi: 10.1371/journal.pone.0202652 (PMC6122789; doi:10.1371/journal.pone.0202652)
Supplement: S6 Fig — The trajectory snapshots of the training-set have been randomly shuffled and the prediction performance on validation trajectories is shown. A notable effect on performance can be observed for improved state class, showing instable learning behavior. Classification precision (purple), recall (red) and F1 (green) as a function of training steps for the three different states (A) improved, (B) no-change and (C) decreased are visualized for CV fold 4. The vertical lines indicate the best performance for each metric based on a moving average with a window-size of 30. (PDF) [file pone.0202652.s006.pdf]

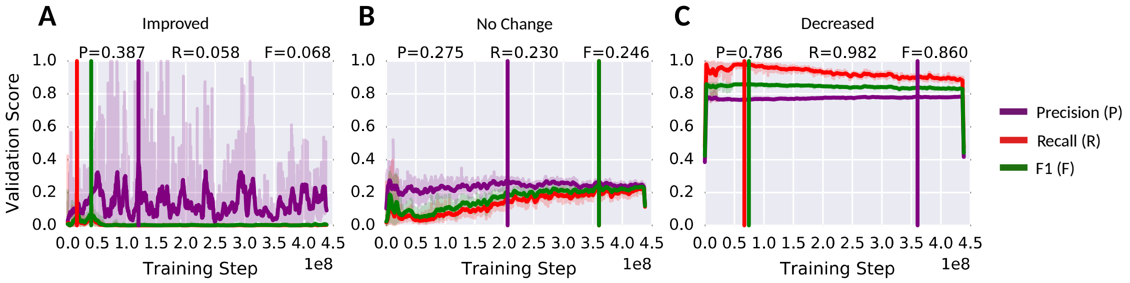


S6 Fig. : Learning curves for randomized trajectory snapshots. The trajectory snapshots of the training-set have been randomly shuffled and the prediction performance on validation trajectories is shown. A notable effect on performance can be observed for improved state class, showing instable learning behavior. Classification precision (purple), recall (red) and F1 (green) as a function of training steps for the three different states (A) improved, (B) no-change and (C) decreased are visualized for CV fold 4. The vertical lines indicate the best performance for each metric based on a moving average with a window-size of 30.
