# Supplementary material for: Predicting improved protein conformations with a temporal deep recurrent neural network
Source: PLoS One. 2018 Sep 4;13(9):e0202652. doi: 10.1371/journal.pone.0202652 (PMC6122789; doi:10.1371/journal.pone.0202652)
Supplement: S1 Table — Table contains the CASP identifier (starting with T0), the CASP refinement identifier (starting with TR), the PDB id of the reference crystal structure and a short description of the protein. (PDF) [file pone.0202652.s007.pdf]

S1 Table. : Protein target overview. Table contains the CASP identifier (starting with T0), the CASP refinement identifier (starting with TR), the PDB id of the reference crystal structure and a short description of the protein.

**Target**	**PDB**	**Description**
TR217/T0817	4WED	Crystal structure of ABC transporter substrate-binding protein from Sinorhizobium meliloti
TR228/T0828	4Z29	Crystal structure of the magnetobacterial protein MtxA C-terminal domain
TR283/T0783	4CVH	Crystal structure of human isoprenoid synthase domain-containing protein
TR759/T0759	4Q28	Crystal Structure of the Plectin 1 and 2 Repeats of the Human Periplakin. Northeast Structural Genomics Consortium (NESG) Target HR9083A
TR760/T0760	4PQX	Crystal structure of a NigD-like protein (BACCAC_02139) from Bacteroides caccae ATCC 43185 at 2.39 A resolution
TR762/T0762	4Q5T	Crystal structure of an atmB (putative membrane lipoprotein) from Streptococcus mutans UA159 at 1.91 A resolution
TR765/T0765	4PWU	Crystal structure of a modulator protein MzrA (KPN_03524) from Klebsiella pneumoniae subsp. pneumoniae MGH 78578 at 2.45 A resolution
TR768/T0768	4OJU	Crystal structure of a leucine-rich repeat protein (BACCAP_00569) from Bacteroides capillosus ATCC 29799 at 2.00 A resolution
TR769/T0769	2MQ8	Solution NMR Structure of De novo designed protein LFR1 1 with ferredoxin fold, Northeast Structural Genomics Consortium (NESG) Target OR414
TR774/T0774	4QB7	Crystal structure of a fimbrial protein (BVU_2522) from Bacteroides vulgatus ATCC 8482 at 2.55 A resolution
TR776/T0776	4Q9A	Crystal structure of a putative GDSL-like lipase (PARMER_00689) from Parabacteroides merdae ATCC 43184 at 2.86 A resolution
TR780/T0780	4QDY	Crystal structure of a YbbR-like protein (SP_1560) from Streptococcus pneumoniae TIGR4 at 2.74 A resolution
TR782/T0782	4GRL	Crystal structure of a autoimmune TCR-MHC complex
TR783/T0783	4CVH	Crystal structure of human isoprenoid synthase domain-containing protein
TR786/T0786	4QVU	Crystal structure of a DUF4931 family protein (BCE0241) from Bacillus cereus ATCC 10987 at 2.65 A resolution
TR792/T0792	5A49	Crystal structure of the LOTUS domain (aa 139-222) of Drosophila Oskar in C222
TR795/T0795	5FJL	Crystal structure of raptor adenovirus 1 fibre head, wild-type form
TR803/T0803	4OGM	MBP-fusion protein of PilA1 residues 26-159
TR810/T0810	5JP6	Bdellovibrio bacteriovorus peptidoglycan deacetylase Bd3279
TR816/T0816	5A1Q	Crystal structure of Archaeoglobus fulgidus Af1502
TR817/T0817	4WED	Crystal structure of ABC transporter substrate-binding protein from Sinorhizobium meliloti
TR821/T0821	4R7S	Crystal structure of a tetratricopeptide repeat protein (PARMER_03812) from Parabacteroides merdae ATCC 43184 at 2.39 A resolution

S1 Table. : Protein target overview of which several MD trajectories were generated

**Target**	**PDB**	**Description**
TR828/T0828	4Z29	Crystal structure of the magnetobacterial protein MtxA C-terminal domain
TR829/T0829	4RQL	Crystal structure of a human cytochrome P450 2B6 (Y226H/K262R) in complex with a monoterpene - sabinene
TR833/T0833	4R03	Crystal structure of a DUF3836 family protein (BDI_3222) from Parabacteroides distasonis ATCC 8503 at 1.50 A resolution
TR837/T0837	5TF3	Crystal Structure of Protein of Unknown Function YPO2564 from Yersinia pestis
TR848/T0848	4R4Q	Crystal structure of RPA70N in complex with C31 H23 Cl2 N3 O6
TR854/T0854	4RN3	Crystal structure of a HAD-superfamily hydrolase, subfamily IA, variant 1 (GSU2069) from Geobacter sulfurreducens PCA at 2.15 A resolution
TR856/T0856	4QT6	Crystal structure of the SPRY domain of human HERC1
TR857/T0857	2MQC	NMR structure of the protein BVU_0925 from Bacteroides vulgatus ATCC 8482
TR862/T0862	5J5V	CdiA-CT from uropathogenic Escherichia coli in complex with cognate immunity protein and CysK
TR868/T0868	5J4A	CdiA-CT toxin from Burkholderia pseudomallei E479 in complex with cognate CdiI immunity protein
TR869/T0869	5J4A	CdiA-CT toxin from Burkholderia pseudomallei E479 in complex with cognate CdiI immunity protein
TR870/T0870	5J5V	CdiA-CT from uropathogenic Escherichia coli in complex with cognate immunity protein and CysK
TR872/T0872	5JMB	The Crystal structure of the N-terminal domain of a novel cellulases from Bacteroides coprocola
TR879/T0879	5JMU	The crystal structure of the catalytic domain of peptidoglycan N-acetylglucosamine deacetylase from Eubacterium rectale ATCC 33656
TR891/T0891	4YMP	Crystal structure of the Bacillus anthracis Hal NEAT domain in complex with heme
TR893/T0893	5IDJ	Bifunctional histidine kinase CckA (domains DHp-CA) in complex with ADP/Mg2+
TR921/T0921	5AOZ	High resolution SeMet structure of the third cohesin from Ruminococcus flavefaciens scaffoldin protein, ScaB
TR928/T0928	5TF2	CRYSTAL STRUCTURE OF THE WD40 DOMAIN OF THE HUMAN PROLACTIN REGULATORY ELEMENT-BINDING PROTEIN
TR944/T0944	5KO9	Crystal Structure of the SRAP Domain of Human HMCES Protein
TR945/T0945	5LEV	Crystal structure of human UDP-N-acetylglucosamine-dolichyl-phosphate N-acetylglucosaminephosphotransferase (DPAGT1) (V264G mutant)
