# Supplementary material for: Predicting improved protein conformations with a temporal deep recurrent neural network
Source: PLoS One. 2018 Sep 4;13(9):e0202652. doi: 10.1371/journal.pone.0202652 (PMC6122789; doi:10.1371/journal.pone.0202652)
Supplement: S2 Table — Each row shows the trajectory name, the cross-validation fold assignment, the number of snapshots per trajectory, the total number of sampling runs and the absolute number of sampled improved, no-change and decreased states. (PDF) [file pone.0202652.s008.pdf]

S2 Table. : Trajectory and cross validation overview for all protein targets. Each row shows the trajectory name, the cross-validation fold assignment, the number of snapshots per trajectory, the total number of sampling runs and the absolute number of sampled improved, no-change and decreased states.

**Traj. Name**	**Fold**	**# Snap.**	**# Traj.**	**I**	**N**	**D**
TR759_dist_rst	0	12008	8	7401	1995	2612
TR759_no_rst	0	11208	8	4260	2231	4717
TR759_point_rst	0	11208	8	5517	1701	3990
TR782_no_rst	0	11208	8	30	171	11007
TR782_point_rst	0	11208	8	149	948	10111
TR810_dist_rst	0	12008	8	18	474	11516
TR810_no_rst	0	11208	8	55	690	10463
TR810_point_rst	0	11208	8	92	7104	4012
TR856_dist_rst	0	12008	8	0	1	12007
TR856_no_rst	0	11208	8	0	3	11205
TR856_point_rst	0	11208	8	0	267	10941
TR869_cm_excl	0	24505	5	18	3077	21410
TR869_cm_min	0	24505	5	3	882	23620
TR869_point_rst	0	11208	8	35	2599	8574
TR891_cm_excl	0	24505	5	4	1895	22606
TR891_cm_min	0	24505	5	28	2622	21855
TR891_point_rst	0	11208	8	12	2230	8966
TR283_dist_rst	1	12008	8	318	1074	10616
TR283_no_rst	1	11208	8	19	531	10658
TR283_point_rst	1	11208	8	0	8	11200
TR780_dist_rst	1	12008	8	4242	1940	5826
TR780_no_rst	1	11208	8	1167	1191	8850
TR780_point_rst	1	11208	8	5729	4651	828
TR837_dist_rst	1	12008	8	1268	886	9854
TR837_no_rst	1	11208	8	1329	1448	8431
TR837_point_rst	1	11208	8	727	1336	9145
TR854_dist_rst	1	12008	8	329	991	10688
TR854_no_rst	1	11208	8	376	1084	9748
TR854_point_rst	1	11208	8	2943	6376	1889
TR879_cm_excl	1	24505	5	0	11	24494
TR879_cm_min	1	24505	5	0	27	24478
TR879_point_rst	1	11208	8	0	40	11168
TR921_cm_excl	1	24505	5	17	6751	17737
TR921_cm_min	1	24505	5	27	9337	15141
TR921_point_rst	1	11208	8	13	6552	4643
TR217_dist_rst	2	12008	8	0	13	11995
TR217_no_rst	2	11208	8	0	15	11193
TR217_point_rst	2	11208	8	583	6864	3761
TR760_dist_rst	2	12008	8	0	8	12000
TR760_no_rst	2	11208	8	0	8	11200
TR760_point_rst	2	11208	8	381	5509	5318
TR786_dist_rst	2	12008	8	2	74	11932
TR786_no_rst	2	11208	8	1	60	11147
TR786_point_rst	2	11208	8	3451	4746	3011
TR816_dist_rst	2	12008	8	1017	488	10503
TR816_no_rst	2	11208	8	1598	1024	8586
TR816_point_rst	2	11208	8	1409	1754	8045
TR862_cm_excl	2	24505	5	422	3323	20760
TR862_cm_min	2	24505	5	2444	4205	17856
TR862_point_rst	2	11208	8	595	2900	7713
TR872_cm_excl	2	24505	5	2201	6844	15460
TR872_cm_min	2	24505	5	1488	4055	18962
TR872_point_rst	2	11208	8	767	3268	7173
TR762_dist_rst	3	12008	8	0	8	12000
TR762_no_rst	3	11208	8	0	8	11200

S2 Table. : Trajectory and cross validation overview for all protein targets.

Traj. Name	Fold	# Snap.	# Traj.	I	N	D
TR762_point_rst	3	11208	8	0	1656	9552
TR765_dist_rst	3	12008	8	11107	551	350
TR765_no_rst	3	11208	8	8259	963	1986
TR765_point_rst	3	11208	8	10719	425	64
TR828_dist_rst	3	12008	8	3	13	11992
TR828_no_rst	3	11208	8	1	15	11192
TR828_point_rst	3	11208	8	7	57	11144
TR833_dist_rst	3	12008	8	2	83	11923
TR833_no_rst	3	11208	8	1	64	11143
TR833_point_rst	3	11208	8	1232	4336	5640
TR928_cm_excl	3	24505	5	0	0	24505
TR928_cm_min	3	24505	5	0	0	24505
TR928_point_rst	3	11208	8	0	0	11208
TR945_cm_excl	3	24505	5	139	13368	10998
TR945_cm_min	3	24505	5	116	10678	13711
TR945_point_rst	3	11208	8	265	7400	3543
TR817_dist_rst	4	12008	8	843	958	10207
TR817_no_rst	4	11208	8	326	576	10306
TR817_point_rst	4	11208	8	73	6538	4597
TR821_dist_rst	4	12008	8	5115	1530	5363
TR821_no_rst	4	11208	8	5235	1458	4515
TR829_dist_rst	4	12008	8	84	425	11499
TR829_no_rst	4	11208	8	41	371	10796
TR829_point_rst	4	11208	8	3213	4337	3658
TR857_dist_rst	4	12008	8	1933	2781	7294
TR857_no_rst	4	11208	8	1734	1944	7530
TR857_point_rst	4	11208	8	1870	3345	5993
TR870_cm_excl	4	24505	5	17	343	24145
TR870_cm_min	4	24505	5	617	2950	20938
TR870_point_rst	4	11208	8	153	1718	9337
TR944_cm_excl	4	24505	5	86	2461	21958
TR944_cm_min	4	24505	5	578	3006	20921
TR944_point_rst	4	11208	8	59	2130	9019
TR769_dist_rst	5	12008	8	2004	1537	8467
TR769_no_rst	5	11208	8	3089	1880	6239
TR774_dist_rst	5	12008	8	1	46	11961
TR774_no_rst	5	11208	8	1	44	11163
TR774_point_rst	5	11208	8	6	1028	10174
TR792_dist_rst	5	12008	8	2815	2383	6810
TR792_no_rst	5	11208	8	3906	2620	4682
TR792_point_rst	5	11208	8	2811	4515	3882
TR795_dist_rst	5	12008	8	373	1367	10268
TR795_point_rst	5	11208	8	1956	7702	1550
TR848_dist_rst	5	12008	8	1	22	11985
TR848_no_rst	5	11208	8	2	53	11153
TR848_point_rst	5	11208	8	77	1541	9590
TR893_cm_excl	5	24505	5	0	121	24384
TR893_cm_min	5	24505	5	0	21	24484
TR893_point_rst	5	11208	8	0	45	11163
TR228_dist_rst	6	12008	8	3073	3563	5372
TR228_no_rst	6	11208	8	4852	2362	3994
TR228_point_rst	6	11208	8	5893	2875	2440
TR768_dist_rst	6	12008	8	50	591	11367
TR768_no_rst	6	11208	8	59	491	10658
TR768_point_rst	6	11208	8	1190	4161	5857
TR776_dist_rst	6	12008	8	0	20	11988
TR776_no_rst	6	11208	8	0	15	11193

S2 Table. : Trajectory and cross validation overview for all protein targets.

**Traj. Name**	**Fold**	**# Snap.**	**# Traj.**	**I**	**N**	**D**
TR776_point_rst	6	11208	8	312	7811	3085
TR783_dist_rst	6	12008	8	10	49	11949
TR783_no_rst	6	11208	8	0	55	11153
TR783_point_rst	6	11208	8	93	1640	9475
TR803_dist_rst	6	12008	8	215	1387	10406
TR803_no_rst	6	11208	8	8	351	10849
TR803_point_rst	6	11208	8	228	1006	9974
TR868_cm_excl	6	24505	5	329	542	23634
TR868_cm_min	6	24505	5	76	503	23926
TR868_point_rst	6	11208	8	592	1293	9323
